# The Malaria Secretome: From Algorithms to Essential Function in Blood Stage Infection

**DOI:** 10.1371/journal.ppat.1000084

**Published:** 2008-06-13

**Authors:** Christiaan van Ooij, Pamela Tamez, Souvik Bhattacharjee, N. Luisa Hiller, Travis Harrison, Konstantinos Liolios, Taco Kooij, Jai Ramesar, Bharath Balu, John Adams, Andy Waters, Chris Janse, Kasturi Haldar

**Affiliations:** 1 Department of Pathology, Northwestern University, Chicago, Illinois, United States of America; 2 Department of Parasitology, Malaria Group, Leiden University Medical Center, Leiden, The Netherlands; 3 Center for Global Health and Infectious Diseases, Department of Biological Sciences, University of Notre Dame, Notre Dame, Indiana, United States of America; 4 Department of Microbiology and Immunology, Northwestern University, Chicago, Illinois, United States of America; Case Western Reserve University, United States of America

## Abstract

The malaria agent *Plasmodium falciparum* is predicted to export a “secretome” of several hundred proteins to remodel the host erythrocyte. Prediction of protein export is based on the presence of an ER-type signal sequence and a downstream Host-Targeting (HT) motif (which is similar to, but distinct from, the closely related Plasmodium Export Element [PEXEL]). Previous attempts to determine the entire secretome, using either the HT-motif or the PEXEL, have yielded large sets of proteins, which have not been comprehensively tested. We present here an expanded secretome that is optimized for both *P. falciparum* signal sequences and the HT-motif. From the most conservative of these three secretome predictions, we identify 11 proteins that are preserved across human- and rodent-infecting *Plasmodium* species. The conservation of these proteins likely indicates that they perform important functions in the interaction with and remodeling of the host erythrocyte important for all *Plasmodium* parasites. Using the *piggyBac* transposition system, we validate their export and find a positive prediction rate of ∼70%. Even for proteins identified by all secretomes, the positive prediction rate is not likely to exceed ∼75%. Attempted deletions of the genes encoding the conserved exported proteins were not successful, but additional functional analyses revealed the first conserved secretome function. This gave new insight into mechanisms for the assembly of the parasite-induced tubovesicular network needed for import of nutrients into the infected erythrocyte. Thus, genomic screens combined with functional assays provide unexpected and fundamental insights into host remodeling by this major human pathogen.

## Author Summary

The parasite *Plasmodium falciparum* causes malaria by replicating inside red blood cells of infected individuals. By exporting many different proteins into the host cell, the parasite changes many of its properties. Knowledge of the identity and function of all the exported proteins will both increase our understanding of the modifications required for parasite survival and provide us with targets that can be inhibited to block the growth of the parasites. Several years ago, a motif within the exported proteins was discovered that allowed them to be exported, which was used to predict the total set of proteins exported to the host cell (the secretome). We show here that the earlier studies have either under- or overestimated the total number of proteins exported into the host cell, and derive a more accurate prediction of proteins exported to the host cell. We validate the predictions by making parasites that express a fusion of predicted exported proteins to the Green Fluorescent Protein (which allows the localization of the protein to be determined visually). This revealed a positive prediction rate of ∼70%. In addition, several proteins were identified that are very likely to play an essential role in infection, with at least one involved in the formation of a structure required for nutrient import.

## Introduction

**Figure 1:**
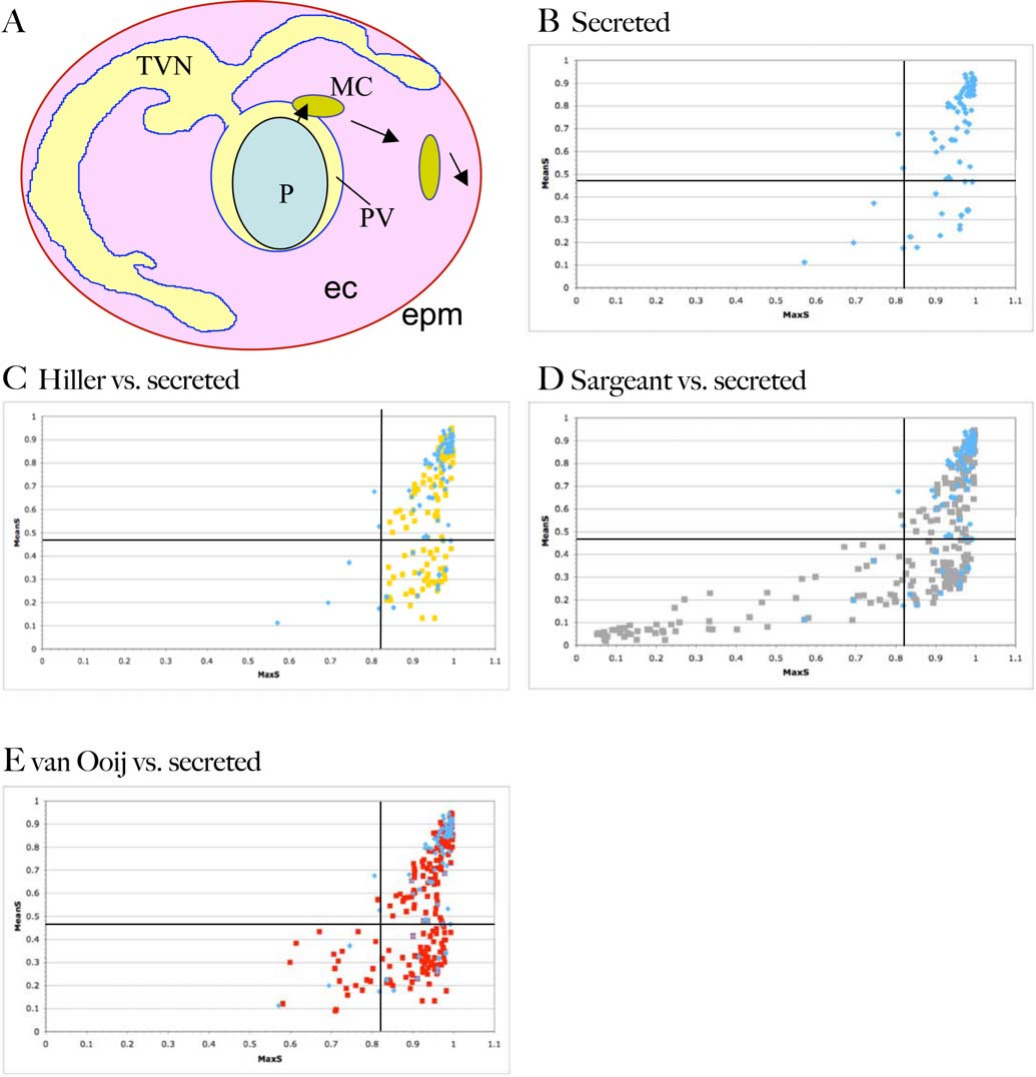
Export of proteins from *P. falciparum* to the erythrocyte. A) Schematic of an erythrocyte infected with *Plasmodium falciparum*. Shown are the parasite (P), the surrounding parasitophorous vacuole (PV), the tubovesicular network (TVN), Maurer's Clefts (MC), the erythrocyte cytosol (ec) and the erythrocyte plasma membrane (epm). Arrows indicate the two steps involved in export of parasite proteins to the erythrocyte, ER-based secretion to the PV followed by HT-dependent transport to clefts (short arrow) followed by cleft movement and subsequent protein delivery to the cytoplasm and membrane of the erythrocyte (long arrow). B) Distribution of MaxS and MeanS values of 82 *P. falciparum* proteins known to enter the secretory pathway. Proteins were identified from the literature and include proteins destined for the apicoplast, apical organelles, parasite plasma membrane, food vacuole, erythrocyte cytosol and erythrocyte plasma membrane (Table S1). MaxS and MeanS values of the N-terminal 100 amino acids were identified using the SignalP 2.0 server. Lines indicate the MaxS and MeanS cut-offs used by SignalP (0.82 and 0.47, respectively). Comparison of the MaxS and MeanS values of the secreted proteins in B) (shown in blue) with the Hiller secretome (yellow) (C), Sargeant secretome (grey) (D), and van Ooij secretome (red) (E). RIFINS and STEVORs are not represented in the plots.

*Plasmodium falciparum* is the protozoan parasite responsible for the most deadly forms of malaria. The symptoms of malaria, which include fevers and chills and can include coma and death, are caused by infection of human erythrocytes by the parasite. After invasion of the erythrocyte, the parasite is contained within a membrane-bound compartment, the parasitophorous vacuole (PV; Fig. 1A). Intracellular parasites induce major changes in several properties of the erythrocyte, including its deformability [1]–[3], permeability of its plasma membrane [4],[5] and its adhesiveness to the endothelium [6]. Underlying these changes are proteins produced by the parasite and exported past the PV membrane (PVM) into the host cell. Examples include ring-infected erythrocyte surface antigen (RESA), which increases the heat-resistance of the erythrocyte [7],[8], and *P. falciparum* erythrocyte membrane protein 1 (PfEMP1), a cell surface adhesin that, together with another parasite protein, Knob-Associated Histidine-Rich Protein (KAHRP), forms knobs on the surface of the erythrocyte that increase the adhesiveness of the infected erythrocyte [9]–[11]. Another important change in the erythrocyte is the appearance of a large membranous network, the tubovesicular network (TVN; Fig. 1A) [12], which plays a role in nutrient import into the parasite. The formation of this import organelle is entirely dependent on the parasite, and previous studies have shown that development of the TVN is linked to nutrient import into infected erythrocytes [12],[13].

For an exported protein to reach the cytosol or membrane of the erythrocyte, it needs to cross two membranes: the parasite plasma membrane and the PVM (Fig. 1A). The first step requires a canonical ER-type signal sequence, while export of many (but not all) proteins from the PV into the erythrocyte depends on a sequence motif referred to as the Host Targeting (HT)-motif [14] [alternatively known as Plasmodium Export Element (PEXEL)] [15] positioned downstream and proximal to the signal sequence. The HT-motif and PEXEL are identified by different algorithms and have slightly different specificities, but recognize the same core sequence (RxLxE/Q/D) [14],[15]. Identification of this export motif allowed prediction of the set of proteins exported into the erythrocyte (the HT-based Hiller secretome and PEXEL-based Marti secretome), and recently an expanded version of the PEXEL-based secretome (Sargeant secretome), identified with the PEXEL-based prediction program ExportPred, was published. All three secretomes are unexpectedly large, containing over 250 proteins each. Excluding the large protein families of RIFINs (165 proteins) and STEVORs (22 proteins, of which only one is synthesized in an individual parasite [16]), the Hiller secretome contains 113 proteins, the Marti secretome 158, and the Sargeant secretome 267 [17]. The overlap of the Hiller and Marti secretomes is only 59 proteins (53% of Hiller set, 37% of Marti set), underscoring the differences in the prediction algorithms.

A large majority of the identified proteins cannot be annotated, and unfortunately, export of nearly all ‘hypothetical’ proteins has not been experimentally verified. In just two cases have full-length fusions of a hypothetical protein with the Green Fluorescent Protein (GFP) been shown to be exported [14],[18]. In six additional cases only the N-terminal region of the proteins was tested [14],[17]. There was no subsequent verification with full-length proteins. Hence the contribution of additional sequences to the export signal was not established. Interestingly, all *P. falciparum* proteins known to be exported into the host cell are species-specific. Hence little is known about the functions of exported proteins shared by all *Plasmodium* species. It is these proteins that are very likely to be involved in the processes that allow the parasites to survive within the erythrocyte, and would make excellent targets for prophylaxis.

Thus identification of the exported proteins and their function will give better insight into the parasite-erythrocyte interaction. We have therefore investigated several parameters of protein export in *P. falciparum* to refine the export prediction. We show that the HT motif-based algorithm PlasmoHT is limited by the identification of ER-type signal sequences in *P. falciparum* and that this represents an important difference with ExportPred. We furthermore identify the putatively exported proteins conserved in other *Plasmodium* species and use this as a high value candidate set to validate the export prediction. Importantly, we find that testing N-terminal regions alone can lead to critical oversight on location of the protein and full-length fusions should be evaluated in order to validate positive predictions. Finally, this study shows that despite the fact that essential genes cannot be knocked out in blood stage *Plasmodium berghei*, insight into biological processes can be attained by utilizing rapid transgene expression. Even so, limitations in the genetic system of *P. falciparum* have not allowed any large scale validation of export predictions, and the testing here improves significantly on previous attempts and enables functional analyses.

## Results

### Analysis of signal sequences of known secreted proteins in *Plasmodium falciparum*

Secreted *P. falciparum* proteins can have recessed signal sequences and thus be difficult to identify with standard signal sequence identification programs such as Signal P [19]. In prior attempts to identify exported proteins Hiller et al. modified SignalP to examine the first 100 residues instead of the default 70 residues [14], while Sargeant et al., abandoned SignalP but instead required the presence of a stretch of 10–25 hydrophobic residues near the N-terminus, separated by a spacer region from the start methionine [17]. However, neither prediction was based on multiple validated sequences. We therefore set out to determine how well SignalP predicts the secretion of known secreted *P. falciparum* proteins in order to obtain a more accurate prediction of secretion. SignalP by default examines the N-terminal 70 amino acids of a protein and determines the most likely cleavage site (represented by the C and Y scores), the maximal signal sequence residues (MaxS), and the average signal sequence residues (MeanS) as measured from the N-terminus to the most likely cleavage site [19]. We plotted the MaxS and MeanS values of 82 proteins known to be transported through the secretory pathway (with destinations as diverse as the apicoplast, parasite plasma membrane, rhoptry and erythrocyte cytosol and membrane, see Table S1) (Fig. 1B). Seventy-six proteins had a score above the MaxS threshold for a signal sequence (0.82), and in many cases the values were close to maximal. However, six (7.3%) had a MaxS score below the threshold, while 21 (25.6%) had a MeanS score below the threshold (0.47); four of these proteins (4.9%) had both a MeanS and MaxS score below threshold. Figure 1B shows that a protein (RESA) with a MaxS score of 0.571 and MeanS score of 0.113 can be secreted. Apparent from Fig. 1B is that as the MeanS scores decrease, the MaxS values become more scattered. Since a lower MeanS score often is indicative of a recessed signal sequence, it is possible that the increased length of the N-terminus before the hydrophobic core of the signal sequence allows for lower MaxS scores. The large percentage of proteins with a low MeanS score is likely a reflection of the prevalence of recessed signal sequences in plasmodial proteins. Thus clearly, while many secreted *P. falciparum* proteins adhere to the same principals of secretion as higher eukaryotes, there may be a degree of flexibility in the signal that allows proteins with lower MaxS scores to be secreted, which in turn leads to under-prediction of secreted proteins by SignalP.

To evaluate whether proteins predicted to be exported to the erythrocyte contained any distinguishing features in their signal sequences, we plotted the MaxS and MeanS scores of the proteins in the previously published Hiller and Sargeant secretomes [14],[17]. Since Hiller et al. used the default 0.82 MaxS cut-off to identify signal sequences, all MaxS scores predictably were above 0.82, but the MeanS scores were scattered within the same range of scores as seen in the group of known secreted proteins (Fig. 1C). The Sargeant secretome is based on the SignalP-independent algorithm ExportPred. As shown in Fig. 1D, like the Hiller secretome, the proteins in the Sargeant secretome fall on the same general curve as the known secreted set, i.e., as the MeanS decreases, the MaxS values become more scattered. However, 50 out of 267 proteins (18%) had signal sequence scores below the lowest experimentally proven functional MaxS score of 0.571 for *Plasmodium*; the fitness of these values for secretion remains to be validated experimentally. Thus one major reason for the different predictions in the Sargeant secretome relative to the Hiller secretome lies in the extremely low SignalP scores the former allows.

To obtain a maximal secretome whose signal sequence predictions were within the range of experimentally verified signals for *Plasmodium*, we re-evaluated the HT-based secretome based on a MaxS cutoff of 0.571. After manual curation this identified an additional 22 putatively exported proteins. We also accommodated recent updates in gene calling in the *P. falciparum* genome to revise secretome predictions obtained by MEME/MAST, the programs used to identify the initial Hiller secretome. Furthermore we utilized a second algorithm, HMMER, to expand the MAST output, which yielded an additional 171 proteins. Combining these proteins with the Hiller secretome (251 proteins) yielded an expanded secretome (van Ooij secretome) of 422 proteins (Table S2). The algorithmic predictions of this secretome were further curated for proteins with MaxS scores between 0.58 and 0.82. No further curation was undertaken based on expression, functional or structural data. Removing the RIFIN and STEVOR family members left 224 proteins (no PfEMP1 family members are present in this secretome since they contain an internal signal sequence and are thus not recognized by Signal P). The van Ooij secretome approaches the Sargeant secretome (267 proteins) in size, but utilizes signal sequence parameters that closely mimic experimental predictions and an HT-motif that is closely linked to the Hiller secretome. The MaxS and MeanS values of the proteins in the van Ooij secretome (omitting the RIFINs and STEVORs) were plotted against each other and were found to follow a similar pattern to the Hiller secretome (Fig. 1D). A comparison of the secretomes and the distribution of MaxS scores is listed in Table S3.

### Exon structure of expanded host-targeted export predictions

It has been noted previously that exported proteins are frequently encoded by genes consisting of two exons [15],[17]. This effect is exaggerated somewhat by the conservation of this exon-intron structure in the *rif* and *stevor* genes, which make up 38.5 and 7.2%, respectively, of the expanded secretome. Even after removal of the *rif* and *stevor* genes, many proteins in the van Ooij secretome are encoded by genes that have this two-exon structure, comprising 69.4% percent of the secretome, and 65% in the Sargeant secretome, with one-exon structure the second-most prevalent in both sets. Distribution of exon structures is shown in Table S4.

### Identification of conserved exported proteins

Previous investigations of exported proteins have focused primarily on the proteins unique to *P. falciparum* or members of large antigenic families unique in other *Plasmodium* species. We were interested in identifying those proteins that are conserved among all *Plasmodium* species because they likely perform functions necessary for the interaction of every *Plasmodium* species with the host erythrocyte. Hence they define the core of the interactions of the parasite with the host cell but remain completely unknown. Our initial analysis of the secretome of rodent malaria parasites *P. berghei*, *Plasmodium chabaudi*, and *Plasmodium yoelii* (Rodent Malaria Parasites (RMP); [14]) and studies by Sargeant et al. [17] on the secretomes of several *Plasmodium* species describe smaller secretomes than those of *P. falciparum*. This could reflect a less complex interaction of those species with the host erythrocyte but is at least in part due to the less complete annotation of the genomes (see Table S5 for examples of changes in annotation of RMP and *Plasmodium vivax* proteins that uncover signal sequence and HT-motifs). Therefore, conserved genes were detected by searching the RMP genomes for orthologues of *P. falciparum* secretome proteins. Since synteny breakpoints often contain species-specific genes [20], we narrowed our search to proteins encoded by genes that had maintained their genomic localization, which additionally aids in identifying those orthologues in which parts of the protein have diverged and thus have a lower score in a BLAST analysis, but are nonetheless bona fide orthologues. On the basis of these criteria, 11 proteins were identified in the original Hiller secretome (Table 1), while the van Ooij secretome contains an additional 18. All syntenic genes were also conserved in *Plasmodium vivax*, indicating the widespread conservation of the genes.

A similar search by Sargeant et al. identified nine putatively exported *P. falciparum* proteins (identified using the PEXEL) that were conserved in *P. vivax* and *P. yoelii* [17]. The overlap of core set predictions by Sargeant et al. and those listed in Table 1 consists of only four proteins (indicated by an asterisk). Two conserved proteins identified by Sargeant et al. but not listed in Table 1 were not recognized as having an HT-motif (underscoring the difference between the predictive algorithms PlasmoHT and ExportPred), while two others did not have a clearly recognizable RMP orthologue in the PlasmoDB database. The remaining protein had a MaxS score of 0.72 and was identified when the MaxS threshold was set at 0.58.

**Table 1:** Syntenic genes encoding predicted exported proteins.

Gene ID Pf	Annotation	Stable *piggyBac* line	Immunoblot-correct size product	maxS	meanS	HT-motif	Export	gene ID Pb	Pb gene deletions	Length of Pf gene cloned
PFC0435w	Hypothetical	Yes	Yes	0.994	0.947	ssifRLLVDtykn	Yes	PB001106.03.0	no mutants obtained	3852/3882 bp
PF10_0177	Surface antigen	Yes	Yes	0.955	0.660	hknkRLLSDtnin	No	PB001047.00.0	no mutants obtained	3027/9496 bp (first exon)
PF14_0607	Hypothetical	Yes	Yes	0.997	0.840	klnnRILFEgsdd	Yes	PB000015.03.0	no mutants obtained	3099/3204bp
PF13_0317	Hypothetical	Yes	No	0.836	0.213	mplwRRFFEgqyt	Not interpretable	PB000056.02.0	no mutants obtained	all
PF13_0090	ADP-ribosylation factor	Yes	Yes	0.973	0.467	ddfiRILYEfrfi	No	PB000767.00.0	no mutants obtained	all
PF13_0218	ABC Transporter	No	Not applicable	0.923	0.133	nyifRAFLDmtkh	Not tested	PB000651.03.0	no mutants obtained	all
PFA0210c*	Hypothetical	Yes	Yes	0.982	0.836	kiksRILKEnkee	Yes	PB300987.00.0	too little sequence information	all
PFC0555c	Hypothetical	Yes	No	0.897	0.583	enmkRILYEwakf	Not interpretable	PB00259.00.0	no mutants obtained	all
PFL0600w*	Hypothetical	Yes	Yes	0.970	0.724	snysRILKEqngk	Yes	PB001041.00.0	no mutants obtained	all
PFL1660c*	Aspartyl protease	Yes	Yes	0.958	0.584	kiikRILSSnslh	No	PB000828.02.0	no mutants obtained	1434/1884bp
PFD0495c*	Hypothetical	Yes	Yes	0.859	0.589	ssfsRIIAEycdt	Yes		no mutations attempted	not applicable

Genes marked with an asterisk were also identified by Sargeant et al. [17]. Several of the orthologues of the 11 proteins did not have an identifiable signal or HT-motif. Close examination of the genomic DNA sequence revealed that in several cases the annotated open reading frame could be extended before the predicted start codon to produce a larger N-terminus that contained both a signal sequence and HT-motif. Several other features of some of the orfs were identified that led us to annotate them slightly differently. These changes are listed in Table S5.

Only two of the eleven syntenic genes, *pf13_0090* and *pfd0495c*, have the classic two-exon structure. In *pf14_0607* the signal sequence is encoded by a small exon, with the HT-motif encoded close to the 5′ end of the second exon, but the entire gene contains twelve additional exons. *pfl1660c* is encoded by five exons, with the signal sequence and the HT-motif, as well as the majority of the protein, encoded by the first exon. The other seven genes consist of a single exon. It is possible that the classic two-exon structure may reflect a mechanism by which *P. falciparum* has been able to convert non-exported proteins to exported proteins through the addition of a small exon encoding a signal sequence and HT-motif. None of the syntenic genes are located near the telomeres. This is not surprising as the telomeres contain many species-specific genes.

These proteins provide a high value set to test the predictive value of each of these secretomes. But since we were interested in testing the prediction of the export motif independent of the SS, we restricted our subsequent analyses to conserved proteins identified in the Hiller secretome (Table 1), which by virtue of using a MaxS cut-off of 0.82 dramatically reduces false positive predictions for recruitment into the secretory pathway, likely to be more prevalent in the more expansive Sargeant and van Ooij secretomes. This assumption is justified by the data in Fig. 1B showing that the vast majority of known secreted *P. falciparum* proteins have MaxS values higher than 0.82.

### Expression of full-length protein fused to a reporter protein is required for optimal assessment of export

**Figure 2:**
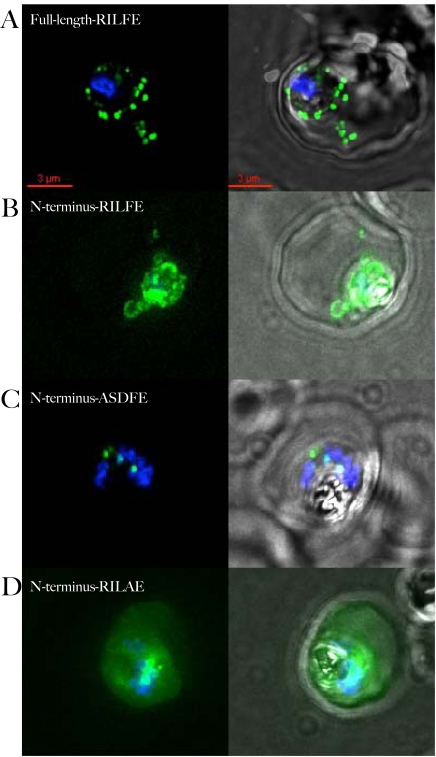
Export of PF14_0607-GFP requires full-length protein. A) Localization of full-length PF14_0607 fused to GFP. The fusion protein is clearly detected in the erythrocyte cytosol. B) Fusion of the wild type N-terminal 89 amino acids to GFP is retained within the PV. Presence of GFP in cytoplasmic loops in the absence of staining in the erythrocyte cytoplasm indicates that the protein is secreted to the PV but not exported into the erythrocyte. C) Replacement of the HT-motif with unrelated sequence leads to retention of the protein inside the parasite. The punctate staining of the mutant fusion protein is reminiscent of apicoplast localization. D) Replacement of phenylalanine at position 4 in the HT-motif with an alanine residue allows the fusion protein to be exported. For each construct, the sequence of the HT-motif, or the sequence replacing it, is shown in the upper left hand corner of the panel. The overlap of GFP and Hoechst staining is shown in the left-hand panels, overlap with the phase contrast image is shown in the right-hand panels. All images are composites of multiple consecutive optical sections.

Sargeant et al. showed that the N-terminal residues of one of the conserved proteins of the Hiller secretome listed in Table 1, PF14_0607, targeted a GFP-fusion to the lumen of the PV, but were not able to promote export to the erythrocyte [17]. This raised the possibility that the leader sequence did not faithfully reflect the export properties of the complete protein. We therefore tested fusions of the full-length gene and the first 89 codons of *pf14_0607* to *gfp*. As shown in Fig. 2A, green fluorescence associated with the full-length fusion was indeed detected in bright punctate spots in the erythrocyte. However, the fusion of the first 89 codons (containing the signal sequence and the HT-motif, but missing the transmembrane domains) was not (or extremely poorly) exported to the erythrocyte (Fig. 2B). Sargeant et al. postulated that the lack of export of the fusion of the N-terminal region of PF14_0607 was due to the presence of a phenylalanine in position 4 of the HT-motif. When this residue was changed to an alanine in the 89 codon-GFP chimera, the protein was indeed robustly exported (Fig. 2D) into the erythrocyte cytosol in an HT-dependent manner (compare Fig. 2C to 2D). These data suggest that a phenylalanine residue at position 4 can indeed hinder export when acting in a short fusion protein that contains just the signal sequence and the HT motif, but in the full-length protein it can be sustained, likely due to structural constraints on the leader imposed by the rest of the protein. Moreover the fact that the mutated 89 codon-GFP fusion is delivered to the erythrocyte cytosol (Fig. 2D), while the full length gene of interest localizes to punctate intraerythrocytic structures (Fig. 2A), suggests that transmembrane and other regions of the protein influence its final destination in the host cell. These data are consistent with our prior studies that have systematically established that while the HT motif is essential for export to the erythrocyte, sequences upstream and downstream provide information [14], [21]–[23] for export resides in overall domain structure that can be conserved across evolutionary distance. The data in Fig. 2 highlight a case where sequences significantly downstream of the HT-motif in the functional protein can likely influence overall structural information needed for HT-motif dependent export. Therefore all subsequent studies were performed with full-length (or nearly full-length) protein fusions (see Table 1).

### Use of the *piggyBac* transposition system for rapid production of transgenic *P. falciparum* with stable genomic integration

Genetic manipulation in *Plasmodium*, while possible, remains a slow process. Successful generation of stable transgenic parasites and initial expansion in amounts required for standard characterization can take 4–6 weeks, while integration into the chromosome via homologous recombination can take months. Therefore most studies have been limited to plasmid-based analysis, which requires continuous drug pressure in culture. Together these limitations have severely handicapped systematic analysis of transgenes in *P. falciparum*.

**Figure 3:**
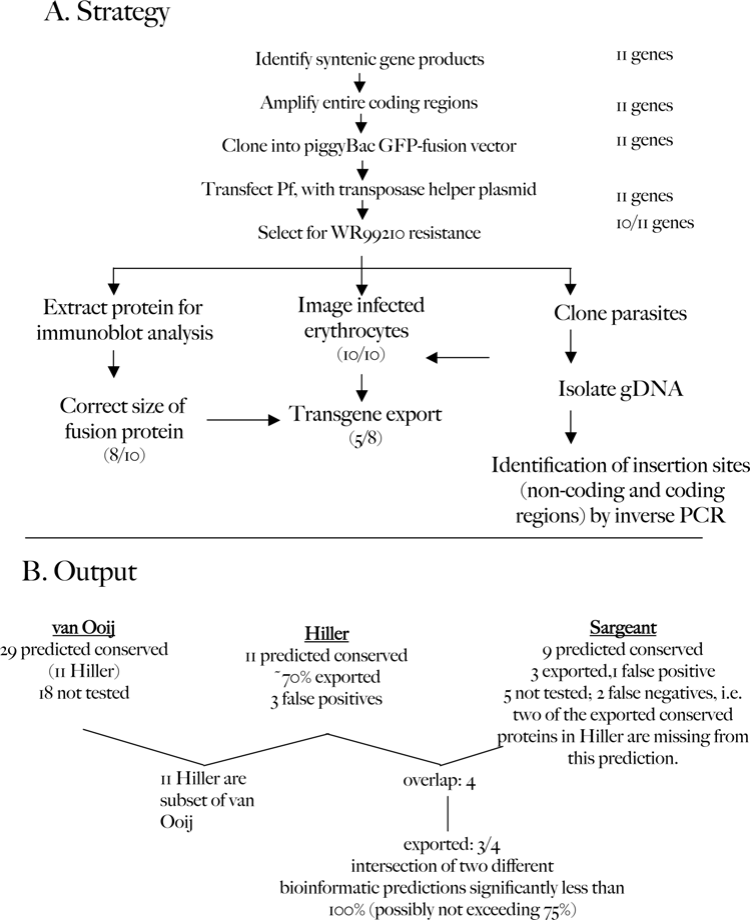
Outline for the validation of secretome predictions. A) Strategy for expression and analysis of syntenic gene products. Numbers on the right-hand side and in parentheses indicate the number of genes that were positive over the number of genes tested. B) Output of data obtained from part A and corresponding analyses in context of Hiller, Sargeant and van Ooij secretomes.

To expedite the production of the stable cell lines expressing a fusion of the syntenic genes with *gfp*, we utilized the *piggyBac* transposition system (see Fig. 3) [24],[25]. This system is based on the integration of specific DNA sequences by the lepidopteran transposase into the sequence TTAA. In the *P. falciparum* strain 3D7, this sequence is present 311,155 times (124,733 times in coding regions), presumably allowing for nearly random integration [25]. To make stably transfected parasites, *P. falciparum* strain 3D7 was transfected with two plasmids, pHTH [25], which encodes the transposase, and a second plasmid that contains a drug marker (human dihydrofolate reductase in this case) and the *gfp*-fusion gene, flanked by the inverted repeats recognized by the transposase. Expression of the transposase then promotes the integration of the inverted repeats into the genome. The plasmid encoding the transposase does not contain a resistance marker and is presumed to be lost during propagation of the parasites. All the transgenic parasites obtained in this study were detected within fourteen days after initiation of drug selection and could be maintained in long-term culture over several months without addition of drug while retaining the *gfp* transgene (Fig. 3).

Integration into genes required for export, resulting in parasites that are no longer capable of protein export, is extremely unlikely, as a subset of exported proteins is likely to fulfill an essential role in the erythrocyte (see below). In principle, integration into essential genes can also occur, but since transposition is likely to occur at many different sites, the selection process after transfection will allow the growth of only those parasites that have no growth defect relative to other transfectants.

### Analysis of full-length fusions of syntenic gene products predicted to be exported to the erythrocyte

While bioinformatics predictions are powerful in identifying candidates for export, they need to be validated in functional assays. We were interested in understanding the HT prediction, independent of the signal sequence prediction, and validated the export of the conserved proteins by making fusions with GFP at the C-terminus. Using the *piggyBac* system, we obtained stably resistant parasites for 10 out of the 11 genes listed in Table 1 within 14 days after transfection; only in the case of PF13_0218-GFP were we unable to obtain stable lines (Fig. 3). Each drug-resistant transformed culture displayed a uniform population of fluorescent parasites such that the export of GFP to the erythrocyte could be ascertained without subsequent cloning of the population. We determined the integration sites for three different clones, and found that transposition had occurred in intergenic sequences as well as open reading frames (Table S6).

Eight of the ten transgenic parasite lines synthesized a fusion protein of the expected size, as judged by anti-GFP immunoblot (Fig. S1). In the two other cases, PF13_0317-GFP and PFC0555c-GFP, a large majority or all of the anti-GFP signal was detected in a band approximately the size of free GFP. Hence PF13_0317 and PFC0555c could not be analysed for export and were excluded from further analysis. The entire analytical procedure is outlined Fig. 3.

**Figure 4:**
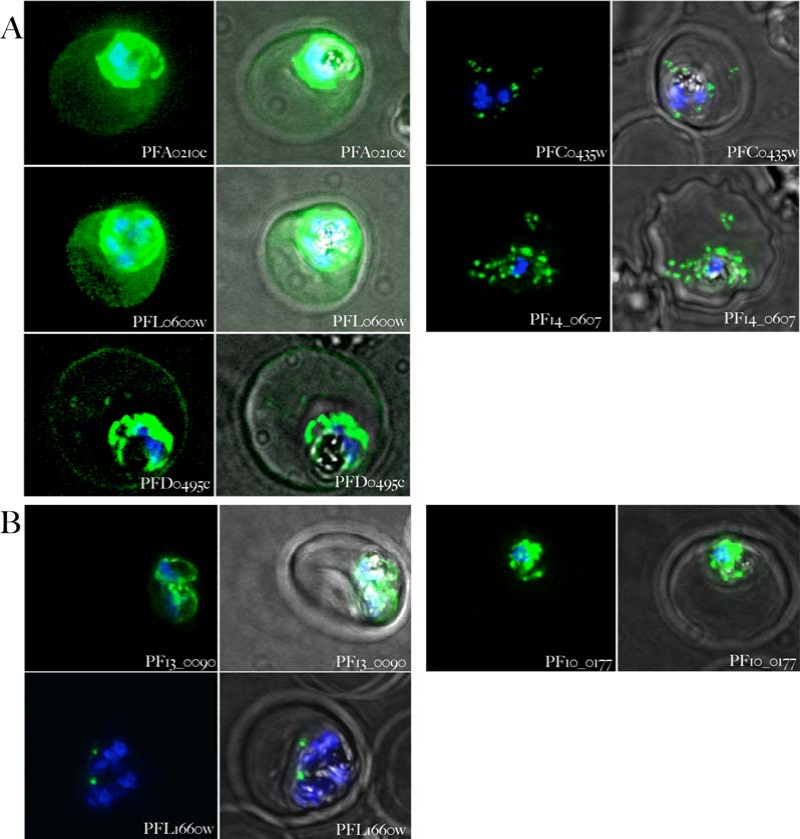
Validation of export of syntenic gene products. A) Syntenic gene products exported to the erythrocyte. Parasites expressing the gene indicated in each panel fused to *gfp* were stained with Hoechst 33342 and examined 18–36 hours after infection. Panels on the left-hand side show the overlap of the Hoechst 33342 staining and the GFP fluorescence, the panels on the right show the Hoechst 33342 staining, the GFP fluorescence and a phase contrast image of the infected cell. B) Syntenic gene products not exported to the erythrocyte. Samples were treated as in A. Notice the different patterns of distribution of the different gene products. All images are composites of multiple consecutive optical sections.

Five GFP-fusions, PFA0210c, PFL0600w, PF14_0607, PFD0495c and PFC0435w, were found to be exported to the erythrocyte (Fig. 4A). PFA0210c-GFP and PFL0600w-GFP were distributed evenly throughout the erythrocyte and were also detected at high levels in the parasitophorous vacuole, which may reflect the high level of expression in the transgene system that uses the strong calmodulin promoter for better visualization of the fusion protein. PFD0495c-GFP, a transmembrane protein was detected at the periphery of the erythrocyte, in intraerythrocytic membranes and the vacuolar parasite. PF14_0607-GFP and PFC0435w-GFP, which also contain transmembrane spanning regions, displayed punctate spots in the erythrocyte as well as closely associated with the PV (Fig. 4A). PFC0435w ends with the sequence DEL but we do not think this C terminal sequence impacts its localization. This is because although xDEL at the C terminus can function as a retention signal for soluble proteins in the lumen of the ER, the ER retention of transmembrane proteins does not involve the xDEL sequence. It should be noted that PFC0435w is not a soluble protein. It is a single pass transmembrane protein with its C-terminus predicted to be on the cytoplasmic face of the ER. Hence PFC0435w is not likely to be an ER retained protein. Three fusion proteins, PFL1660c, PF10_0177 and PF13_0090, were not detected within the erythrocyte (Fig. 4B). The lack of export of PFL1660c-GFP is particularly surprising since it is also predicted to be exported by ExportPred [17]. The protein is annotated to be an aspartyl protease, was detected in small structures inside the parasite (possibly the apicoplast; Fig. 4B) and its N-terminus was recognized as an apicoplast targeting sequence by the PATS program [26]. Residue 5 of its HT-motif is a serine, which is an unusual amino acid for this position, and may be part of the reason the protein is not exported. The perinuclear staining of PF10_0177-GFP indicated the protein was not exported. It should be pointed out that for technical reasons, this fusion contained only the N-terminal 1015 residues (out of 3162, encompassing the first exon only). Although we think it unlikely, it is formally possible that lack of downstream sequences may have compromised export mediated by its HT motif. However, the two exons of the orthologues of PF10_0177 in RMP and *P. vivax* are annotated as two separate genes, in which case the entire gene was fused to *gfp*. PF13_0090, which is annotated as a possible ARF family member, also appeared to associate with the parasite, with no detectable export to the erythrocyte (Fig. 4). When the ability of the N-terminal region of PF13_0090 to direct secretion was tested by fusing the first 59 codons to *gfp*, the resulting fusion protein accumulated inside the parasite rather than in the PV (data not shown), suggesting that despite its high MaxS score, the predicted SS may not support recruitment into the secretory pathway.

In summary, the data in this section suggest that six of the eight tested conserved proteins contain bona fide signal sequences that enable their recruitment into the secretory pathway. Further, 5 of the 8 could be detected exported to the erythrocyte (either diffuse or in punctuate structures) when fused to GFP. Thus we could confirm export with a prediction rate of ∼70% (Fig. 3B). When the overlap of the Hiller, van Ooij and Sargeant secretomes is considered (Fig. 3B), only three of the four conserved proteins predicted to be exported by all three secretomes are exported, while one (PFL1660c) may be transported to an internal secretory destination such as the apicoplast. So while the rate of successful prediction increases when the intersection of two predictions is considered, the success rate is still below 100% (and likely not to exceed to 75%), indicating that there may be additional determinants that are currently not recognized by *in silico* prediction programs. Our data also show that two of Sargeant's predictions of conserved exported proteins [17] were false negatives. One of these proteins (PF14_0607) is excluded from ExportPred because the N-terminal region is not able to promote export, even though the full-length protein is exported. In the other protein (PFC0435w) the HT-motif is separated from the signal peptide cleavage site by 68 residues, which likely exceeds the length probability of the spacer region between the N-terminal hydrophobic region and the HT motif (PEXEL) allowed by ExportPred.

### Syntenic genes encoding exported proteins are essential

In order to learn more about the role of the conserved proteins during the intraerythrocytic cycle, we attempted to delete the genes encoding nine of the eleven of these proteins in the *P. berghei* system [27],[28]. The deletions were attempted twice for each gene, and in no case were mutant parasites obtained, while transfections performed concurrently with unrelated deletion plasmids did produce mutant parasites. The inability to delete these genes strengthens the belief that these genes encode proteins essential for the growth of the parasite within the erythrocyte, which is not unexpected considering the high degree of conservation of these genes. Results of the deletions are listed in Table 1.

### Functional investigation of a conserved protein reveals its involvement in an essential nutrient import pathway

Most exported proteins have no *in silico* annotatable features. However transgenic parasites expressing GFP fusions potentially provide powerful reagents to enable functional characterization. This is especially so when the transcriptional profile of the gene of interest mimics the largely constitutive activity of the *cam* promoter with peak expression from 24–40 h of infection (Fig. S2). Many secretome genes are highly stage specific and show peak expression times at segmenters and early ring stages. However the expression profile of PFC0435w peaks at 24–40 h of infection and closely parallels that of *cam* in the second half of the asexual life cycle (Fig. S2). We confirmed expression of the fusion of the transgene at 24, 36 and 42 h of infection demonstrating that a single fusion product is detected in rings and becomes prominent in the trophozoite and schizont stages (Fig. S3). These data confirmed that expression of PFC0435w-GFP closely mimicked that predicted for endogenous PFC0435w.

**Figure 5:**
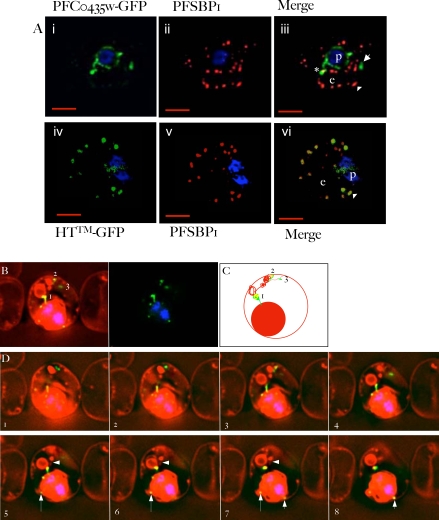
PFC0435w is a junctional protein of the TVN. A) Relative distribution of PFC0435w-GFP (i) or HT^TM^-GFP (ii) and Maurer's clefts marked by PfSBP1 (ii, iv) in *P. falciparum* infected erythrocytes.The cells were also stained with Hoechst 33342 (blue) to visualize the DNA of the parasite. Panels i, ii and iv, v are single optical sections, whose merge is shown in iii and vi respectively. Scale bar, 2 µm. B–D) Association of PFC0435w-GFP with membrane buds and TVN structures B. Zero degree projection of an erythrocyte infected with a PFC0435w-GFP-expressing parasite stained with Rhodamine B. The numbers indicate the large circular TVN structure (1), and PFC0435w-GFP associated with other parts of the TVN (2 and 3). C) Cartoon representation of the infected cell in panel B. D) Optical sectioning of the infected erythrocyte shown in panel B. Distance between sections is 0.2 µm. Note the progression of PFC0435w-GFP staining from the PVM (sections 1–4) to the circular Rhodamine B-stained structure in the erythrocyte (sections 4–8). Arrows indicate PVM buds colocalizing with PFC0435w-GFP. Arrowhead indicates TVN connections between two large loops.

We next examined transgenic lines expressing PFC0435w-GFP at the trophozoite stage by fluorescence microscopy. We confirmed that the fusion was detected in the periphery of the parasite as well as intraerythrocytic structures. However exported PFC0435w-GFP (Fig. 5Aiii, arrow) did not colocalize with Maurer's clefts (Fig. 5Aiii, arrow head), major intraerythrocytic structures implicated in protein export to the erythrocyte membrane. Clefts are flat lamellar membranes exported from the parasite to the erythrocyte and our recent data suggest that they are targeted by the HT motif as conduits for protein export to the cytoplasm and membrane of infected erythrocyte [29]. It is possible that colocalization between one cleft (of ∼10) and PFC0435w-GFP in the trophozoite stage in Fig. 5Aiii (see asterisk) may reflect transport of the fusion through a cleft, en route to a distinct intraerythrocytic destination. A chimeric gene containing just the HT motif and a transmembrane domain expressed by the *cam* promoter, drives efficient export to the clefts (Fig 5Avi, arrow heads), confirming that expression of a transgene via the *cam* promoter does not preclude its quantitative localization to clefts. Together these data suggested PFC0435w-GFP was not a major resident protein of clefts.

We were next interested in determining the relative distribution of PFC0435w-GFP in a tubovesicular import pathway that appears to be distinct from clefts [12]. To do this we determined the distribution of PFC0435w-GFP in relation to Rhodamine B, a fluorescent dye that does not freely diffuse into erythrocytes but is actively internalized into infected erythrocytes by the TVN (Bhattacharjee and Haldar, unpublished data). As shown in Fig. 5B–D, Rhodamine B fluorescence accumulated to a high level within the parasite, as well as in tubovesicular structures that extend from the erythrocyte membrane to the parasite. Remarkably, PFC0435w-GFP could be detected in discrete localized regions of the TVN. The GFP-tagged protein apparently connects the vacuolar parasite to a large membrane loop of the TVN. Analysis of different optical sections from an infected cell confirmed that the protein appeared to form a bridge between the vacuolar parasite and the intraerythrocytic loops; curiously this bridge itself was relatively poorly stained with Rhodamine B (Fig. 5B, D), suggesting it did not retain significant amounts of the internalized probe. PFC0435w-GFP was also detected in junctions between TVN structures closer to distal regions of the TVN near the erythrocyte membrane (Fig. 5B, D and schematized in 5C). These data provide the first direct evidence of a parasite protein quantitatively located at the junction of the TVN and the PVM, and we therefore renamed the protein TVN-junction protein 1 (TVN-JP1). TVN-JP1-GFP was also seen at points where the PVM formed small buds (see arrows in Fig. 5D), suggesting it may define junction sites of loop formation at the PVM.

**Figure 6:**
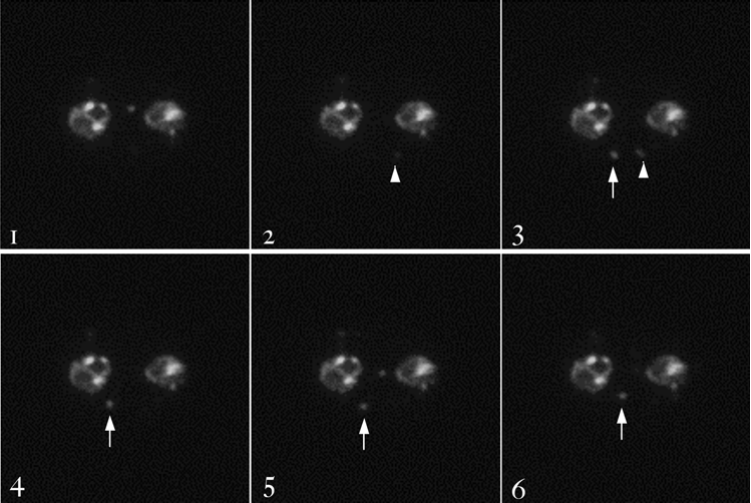
Still images from Video S1. Images shown are the first six of the video (31 total), taken 1 second apart, with an exposure time of 180 msec. Note the appearance and disappearance of a vesicle from the plane of focus, as indicated with the arrow head, and the slower movement of a vesicle within the plane of focus, indicated by the arrow.

The TVN develops during ring to trophozoite development (which occurs approximately 24 hrs after invasion of the erythrocyte, midway in the 48 hr developmental cycle). To further investigate the function of TVN-JP1 in TVN assembly, we examined its distribution in rings. At this stage TVN-JP1 was present in small vesicles that moved rapidly (in seconds) in the host cell cytosol (see Video S1, Fig. 6). This was in sharp contrast with immobile GFP junctions connecting large membrane loops in association with the mature TVN in trophozoites. The movement of the vesicles appeared random, moving away from or towards the parasite equally, and no build-up of the protein at the periphery of the infected cell was detected, indicating that the vesicles did not fuse with compartments at the erythrocyte periphery. However movement of one vesicle (above left-hand cell in Video S1) was highly restricted, suggesting that it may be attached in the erythrocyte. Remarkably, we also detect a membrane connection between this vesicular structure and the parasite, as indicated by the presence of GFP fluorescence spanning from this vesicle to the parasite. This type of vesicle movement has been detected previously in the form of acridine orange-labeled vesicles, which were also detected primarily during the ring stage [30] but their function was unknown. Our data suggest that they are precursors to TVN assembly. The acridine orange-labeled vesicles were detected in wild-type *P. falciparum*, making it unlikely that the appearance of PFC0435w-containing vesicles is an artifact resulting from overexpression of the protein. Together, these data suggest that export of highly mobile vesicles containing TVN-JP1 is an early step in the formation of the TVN. Since TVN-JP1 domains in the erythrocyte are immobile in trophozoites, anchoring of these vesicles and membrane connections between them and the vacuolar parasite are likely to precede sphingolipid-dependent budding of large vesicles and loops originally described as the first step of TVN biogenesis [31]. Thus although TVN-JP1 does not stain large domains of the TVN, its export is nonetheless expected to be important in erythrocyte remodeling and for proper development of the TVN. Our studies establish that rapid genetic methodologies enable identification of genes and mechanisms linked to formation and function of the *P. falciparum* TVN, suggesting they provide new targets for prophylaxis.

## Discussion

In this study we have provided empirical testing of the prediction of protein export from the malaria parasite *P. falciparum* and validation of the export prediction for a set of 11 proteins conserved throughout the genus *Plasmodium*. This is the first demonstration of export of proteins conserved in several species of *Plasmodium*. Relative to the size of the entire secretome this is a relatively small set of proteins to test, but their conservation across species confirms they are high-value determinants. Due to the limitations of genetic manipulation in *P. falciparum*, this kind of genome-wide analysis required utilization of a robust transgene expression system such as *piggyBac*, which shortened the time required for establishing stably transfected parasites and had a very high success rate (10 out of 11 genes could be investigated). Since we selected from a set that had the highest MaxS scores, we maximized chances that these proteins are recruited to the secretory pathway (although the putative ARF is likely not). This increases the ability to evaluate the predictive value of the HT motif in mediating translocation beyond the PV to the erythrocyte Extrapolating the positive prediction rate of ∼70% of the tested proteins to the entire secretome predicts that well over 200 proteins will be exported, confirming original projections that host remodeling is a highly complex process. Nonetheless, due to the difficulty in predicting signal sequences in *Plasmodium*, we expect that in the more expansive secretomes (such as the van Ooij and Sargeant secretomes), the positive prediction rate will be lower. As pointed out previously, two of the proteins shown to be exported were not recognized by the ExportPred algorithm, likely due to a relatively large number of residues separating the HT motif and the signal sequence (PFC0435w) and additional information in the remainder of the proteins (PF14_0607), indicating that parts of the algorithm limit the prediction of the secretome. It is difficult to explain fully why a fusion containing only the 90 N-terminal residues of PF14_0607 remained in the PV while the full-length proteins is exported, other than that the remainder of the protein must aid in exporting the protein across the PV. It is interesting to note that the N-terminal sequence of PF14_0607, and that of 66 other secretome proteins, was recognized by the apicoplast-targeting prediction program PATS [26] (Table S2). This underscores the difficulty of predicting transport when multiple transport signals are present. How overlapping targeting sequences are resolved remains unclear.

Only 11 proteins of the original Hiller secretome were conserved in the RMP and *P. vivax*. This constitutes 9.7% of the total secretome (without the RIFINs and STEVORs), a surprisingly low number. In the Sargeant secretome the percentage of conserved proteins is even lower, 3.3%, while in the van Ooij secretome it is 12.9%. Since we found that the annotation of the orthologous genes of exported proteins often did not include the 5′ region (which encodes the signal sequence; Table S5), it is not yet possible to determine a complete secretome for the RMP or *P. vivax* to provide a direct comparison of the secretomes and identify all RMP or *P. vivax*-specific exported proteins. Even so, a large number of proteins in the *P. falciparum* secretome are species-specific, making it likely that they are important for *P. falciparum*-specific symptoms but possibly not survival of the parasite within the erythrocyte. Indeed, as shown in Table 2, none of the published deletions of genes encoding exported proteins, all *P. falciparum*-specific, are lethal to the parasite. Thus *P. falciparum* contains species-specific mechanisms for sequestration involving PfEMP1 and KAHRP [10], stabilization of the cytoskeleton through RESA [7],[8] and MESA [32], among other proteins, as well as formation of intraerythrocytic structures the Maurer's Clefts, by SBP1 [33] and MAHRP [34], but these processes are not required for the growth of the parasites in culture. Since all the exported proteins studied to date are *P. falciparum*-specific, little is known about the functions shared by all *Plasmodium* species. It is likely that the conservation of the exported proteins, along with the likely function in host cell remodeling, will make these proteins essential factors for parasite survival with the host cell. Consistent with this is the finding that none of the genes encoding the conserved exported proteins could be deleted in *P. berghei*, indicating that they are indeed required for parasite growth (Tables 1, 2).

**Table 2:** Essential nature of known exported proteins.

Non-essential	Not tested	Essential
**PfEMP1**	**MAHRP**	EVP1 (PFD0495c)**
**STEVOR**	**RIFIN**	TVN-JP1(PFC0435w)
**PfMC-2TM**	**FIKK kinases**	PF14_0607
**RESA**		PFA0210c*
**HRP I (KAHRP)**		PFL0600w
**HRP II**		
**HRP III**		
**SBP I**		
**PFE0055c**		

Validated exported proteins for which data are available are categorized as “non-essential”, “not tested”, or “essential” [8], [16], [33], [41]–[44]. Proteins in bold are *P. falciparum-specific*. ^*^) The lack of sequence information for the *P. berghei* orthologue of *pfa0210c* did not allow us to attempt to delete the gene, however, the conserved nature of the gene and the export of the protein product, similar to *pfd0495c*, *pfc0435w* and *pf14_0607*, make it highly likely that the protein is essential. ^**^) Essential nature of *pfd0495c* was established separately (Tamez and Haldar, unpublished).

**Figure 7:**
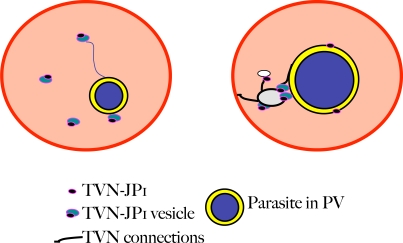
Model for action of conserved exported protein TVN-JP1 in the formation of the TVN. In early (ring) stages (left-hand panel), TVN-JP1 is exported and present in small vesicles, which may emerge from the PVM and remain tethered to the PVM. At later stages (right-hand panel), TVN-JP1 is present in a neck structure, connecting the loops in the TVN to the PVM, as well as at sites interconnecting parts of the TVN, and on buds of the PVM.

The results presented here suggest that at least one conserved exported protein is involved in formation of the TVN, an organelle of nutrient uptake. At the time of its original identification [12],[31], only a parasite sphingomyelin synthase activity was known to be important for TVN synthesis, but no report has described the localization of this sphingomyelin synthase activity. Members of the *P. falciparum* protein family ETRAMPS as well as the *P. berghei* protein UIS4 have been detected on vesicular elements budding off the PVM of blood stage and liver stage parasites, respectively [35]–[37], but since they are detected primarily on the vacuole, they are not thought to be TVN resident proteins or have TVN-specific functions. This study reveals TVN-JP1 as the first TVN-specific protein marker. The distribution of the protein, in a bridge structure between large intraerythrocytic loops and the PVM, could be indicative of a structural role, which would be congruent with the finding that the protein is initially found in rapidly moving vesicles in the erythrocyte cytosol. The evidence that the TVN begins as small, mobile structures in the erythrocyte cytosol is highly unexpected since the mature TVN organelle is a relatively immobile structure in the trophozoite-infected erythrocyte as are the TVN-JP1 junction and large loops between these junctions (Fig. 6,7). The localization of TVN-JP1 at sites of PVM budding in trophozoites may reflect sites of budding that contribute to TVN development even at these later stages of growth (summarized in Fig. 7). Considering the size of the TVN within the erythrocyte cytosol and the functions in protein import it plays, there are undoubtedly more proteins involved in the formation of this organelle. The total number of conserved proteins that could be involved in TVN function and/or formation based on Hiller, Sargeant and van Ooij secretomes are expected to range from ∼10–29. The total number of *P. falciparum*-specific proteins associated with TVN function is presently difficult to predict.

The approach of a genome-wide screen for exported proteins combined with application of several criteria consecutively (conservation in other *Plasmodium* species, validation of export, timing of expression and localization data) has identified possible functions for a protein that was beyond annotation by *in silico* methods. By altering the criteria for selection, it should be possible to uncover the role of other pathogenic exported proteins of hypothetical function and their contribution to intracellular survival and pathogenesis.

## Materials and Methods

### Parasites

*Plasmodium falciparum* parasites used in this study were of the 3D7 lineage and maintained in human erythrocytes, blood type A+, in RPMI-1640 medium supplemented with 91.9 µM hypoxanthine, 11 mM glucose, 0.18% sodium bicarbonate and 10% human serum (cRPMI). *Plasmodium berghei*, strain ANKA, parasites were maintained in Swiss mice.

### Transfection

*P. falciparum* transfections were performed as described by Wu et al. [38] with modifications described by Deitsch et al. [39]. Briefly, erythrocytes were loaded with 100 µg of plasmid DNA containing the transgene, and where necessary, 100 µg of plasmid containing the *piggyBac* transposase (pHTH), by electroporation using a BioRad GenePulser set at 0.310 kV and 950 µF. Transfected erythrocytes were washed three times with cRPMI and immediately mixed with Percoll-purified infected erythrocytes to a parasitemia of 1–5%. Drug selection was initiated by addition of WR99120 (Jacobus Pharmaceuticals, Princeton, NJ) to 2.5 nM 72–96 hours after infection. The medium was changed every other day until parasites were detected by Giemsa staining. Most transfected parasites could be detected within two weeks after drug selection. The *P. falciparum* strain expressing the HT^TM^-GFP fusion is described in Bhattacharjee et al. [29].

*P. berghei* transfection was performed according to published methods [27],[28]. Briefly, 5–8 ml blood was extracted from an infected Wistar rat at a parasitemia of <3%. Blood was mixed with an approximately equal volume of RPMI with L-glutamine and HEPES, supplemented with 0.085% NaHCO3 and 25% fetal calf serum (culture medium) with 0.3 ml heparin stock solution (200 I.U./ml) and spun down. Cells were resuspended in 150 ml culture medium and allowed to mature to the schizont phase overnight by incubation at 37°C in an atmosphere of 5% O_2_, 5% CO_2_, 90% N_2_. Mature parasites were harvested by density centrifugation on Nycodenz and subsequently transfected with 5–10 µg linearized plasmid DNA using the AMAXA device.

### Signal peptide prediction

To search for the presence of an ER-type signal sequence, proteins were analyzed using the SignalP-NN algorithm from the SignalP V2.0.b2 program (http://www.cbs.dtu.dk/services/SignalP-2.0/) [19]. This algorithm was trained on eukaryotic sequences and the input protein sequences were truncated at amino acid 100.

MeanS scores and MaxS scores for each sequence were plotted against each other. In our previously described secretome, all sequences with a maximum S-score above the default cutoff of 0.82 determined by the SignalP-NN software, were considered to have a signal peptide. For the revised secretome set defined in this paper, a MaxS-score of 0.58 was used as the discriminating factor for secretion signals.

### Bioinformatics

The HMMER software suite (http://www.psc.edu/general/software/packages/hmmer/manual/main.html) was used for making a hidden Markov model (HMM). The initial model was built using a training set that consisted of five proteins known to be exported to the erythrocyte (GBP130, PfEMP2, PfEMP3, HRP1, HRP2), and this model was used to search a database of 3D7 proteins with a signal sequence predicted using SignalP 2.0 default maximum S-score as a discriminating factor [14]. The proteins identified from this search were used as a new training set to create the final HMM. This model was used to screen all proteins predicted to have a signal sequence according to the SignalP maximum S-score cutoff of 0.58 (see Signal Sequence Prediction section below) for sequences that contain the HMM.

### Identification of syntenic genes

The chromosomal position of all 3D7 sequences in the original PlasmoHT set [14] were compared with the chromosomal position of their orthologues in the rodent malaria *P. berghei* and *P. yoelii* described in Kooij et al. [25]. e-values were used as a guide to predict functional class and synteny was used to select the functional orthologues.

### Plasmids

The syntenic genes were amplified with the primers listed in Table S7 (which also list the amount of the gene amplified) using *P. falciparum* 3D7 genomic DNA as template. The resulting DNA fragments containing *pf13_0317, pf13_0090*, *pf13_0218*, *pfa0210c*, *pfc0555c*, *pfl0600w*, and *pfd0495c* were digested with the indicated restriction enzymes and cloned into pBLD70 to make a fusion with *gfp*. pBLD70 was made by cloning GFP mut2 followed by an XhoI site into pBluescript using the NheI and SacI sites. This fusion was liberated with XhoI and cloned into XhoI-digested pBLD194. *pfc0435w*, *pf10_0177*, *pf14_0607*, and *pfl1660c* were amplified further using AttB1 and AttB2 primers to attach full-length Att-sites on the DNA fragments, and cloned into pDONR and subsequently transferred to the GFP-fusion expression vector pBLD194 using the GATEWAY technology (Invitrogen, Inc, Carlsbad, CA).

### Truncated and mutated *pf14_0607*

To make a truncated version of *pf14_0607*, the 5′ 89 codons were amplified using the primers listed is Table S7 using plasmid pBLD201, the *pf14_0607* full-length expression vector described above, as template. The resulting DNA fragment was cloned into pDONR and subsequently moved to the GFP-expression vector pBLD194. To make the F-A mutation or the replacement of the HT-motif, the 89 codon 5′ region was amplified in two parts using the primers listed in Table S7 (which include the intended mutation). The resulting fragments overlapped by 47 bp (including the introduced mutation), and were mixed with the 5′ upstream and 3′ downstream primers in a PCR to produce the entire 89-codon fragment that contains the intended mutation. This fragment was again amplified with the AttB primers to introduce full AttB sites and then cloned into pDONR and subsequently pBLD194 using the GATEWAY technology.

### Deletion vectors

Vectors for the targeted deletion of the syntenic genes in *P. berghei* were made as follows. A region of DNA upstream and downstream of the target gene was amplified, with each fragment in the range of 650–1100 bp (with exception of the region upstream of PB000767.00.0, which was approximately 3 kb), using 100 ng *P. berghei* genomic DNA as template. Primers used for amplification are listed in Table S7. The amplified DNA fragments were inserted into the pDONR vector using the GATEWAY technology. The upstream fragments were released from the resulting vectors using SacII and SpeI and cloned into corresponding sites in pL0001. Subsequently the corresponding downstream regions were cloned into the resulting vector with HindIII and KpnI to form the final deletion vector with the upstream and downstream regions flanking the hDHFR resistance cassette. For transfection the plasmids were linearized by digestion with KpnI.

### *piggyBac*-based integration

To obtain stable parasite lines with integrated copies of the transgene, parasites were transfected as described above with 100 µg of vector containing the transgene and 100 µg of vector containing the transposase, pHTH [24],[25]. To identify the sites of integration, genomic DNA was extracted from ∼2–9×10^8^ cloned parasites. Parasites were extracted from infected erythrocytes by lysis with 0.05% saponin in PBS. Parasites were pelleted and lysis was repeated. Parasites were washed with PBS twice and frozen at −80°C. Genomic DNA was isolated by standard procedure. Briefly, parasites were resuspended in lysis buffer (10 mM Tris, 20 mM EDTA, 0.5% SDS, 25 µg/ml Proteinase K) and incubated at 37°C for 3 hours. The lysate was extracted once with phenol and once with chloroform and treated with RNase A (20 µg/ml) for 15 minutes at 37°C. The DNA was subsequently extracted twice with phenol-chloroform and precipitated with ethanol. One µg of DNA was digested with Sau3AI overnight, ethanol precipitated, and self-ligated in a 100 µl-reaction overnight. The ligated DNA was precipitated with ethanol and resuspended in 20 µl. Of this, 1 µl was used for PCR using previously described primers [25]. The resulting DNA fragments were cloned into pGEM-T and sequenced using M13 forward and M13 reverse primers.

### Cloning of parasites

Parasites were cloned by limiting dilution. Parasitemia of cultures was determined by Giemsa staining and parasites diluted to 3 parasites/ml in cRPMI. Of this dilution, 100 µl was added to one well of a 96-well plate, and 10 µl of 50% human erythrocytes and 90 µl of cRPMI were added. Medium was changed and erythrocytes added every four days. Parasites were detected approximately 18 days after dilution.

### Fluorescence microscopy

Parasites were prepared for microscopy as described [21]. Briefly, ∼3×10^8^ erythrocytes infected at a parasitemia of 1–10% were spun down and resuspended in 1 ml PBS with 11 mM glucose containing 10 µg/ml Hoechst 33342 and left at room temperature for five minutes. The cells were washed twice with 3 ml PBS-glucose and resuspended to a parasitemia of ∼30%. A 3 µl aliquot was placed on a slide and covered with a coverslip, which was sealed with nail polish. The sample was viewed on an Olympus IX inverted fluorescence microscope and images were collected with a Photometrix cooled CCD camera (CH350/LCCD) driven by DeltaVision software from Applied Precision Inc. (Seattle, WA).

For immunofluorescence staining, we followed the protocol of Apodaca et al. [40]. Briefly, cells were deposited on poly-L-lysine coated coverslips for thirty minutes at 37°C in PBS containing 11 mM glucose. Cells were washed twice with PBS and fixed with 1% formaldehyde in PBS for ten minutes, then quenched with 50 mM ammonium chloride in PBS for ten minutes. Then the cells were washed, permeabilized with 0.05% saponin in PBS and blocked with 0.7% fish skin gelatin, 0.01% saponin in PBS (FSP) for thirty minutes at 37°C. Primary antibody against SBP1 (LWL) and GFP diluted in FSP were added and the cells were incubated for one hour at 37°C. Cells were washed three times with FSP before addition of secondary antibody (goat anti-mouse TRITC and goat anti-rabbit FITC) and incubation at 37°C for one hour. Cells were then washed three times with FSP, twice with PBS, once with PBS containing 0.01% saponin, once with PBS, once with PBS containing 0.1% Triton X-100 and once with PBS. Cells were then fixed again with 4% formaldehyde in 0.1 M sodium cacodylate for thirty minutes. The cells were washed once with PBS, then stained with 10 µg/ml Hoechst 33342 in PBS for five minutes. The cells were washed two more times with PBS before being mounted on glass slides on a drop of DABCO and sealed with nail polish. Slides were stored at −20°C.

### Immunoblotting

Parasites were harvested from erythrocytes with 0.05% saponin as described above and frozen at −80°C. In some cases the parasites were resuspended directly in SDS-PAGE loading dye, boiled, and an equivalent of 1–5×10^7^ parasites was loaded on an SDS-PAGE gel after boiling. In other cases the parasites were resuspended in 100 µl RIPA buffer, incubated on ice in the presence of protease inhibitors. An equivalent volume of SDS-PAGE loading dye was added, the samples were boiled and the samples run as above. Proteins were transferred to nitrocellulose. The resulting blot was blocked with 5% milk in PBS with 0.05% Tween-20 (PBS-T) for one hour at room temperature or at 4°C overnight, and incubated with anti-GFP antibody (Santa Cruz, Molecular Probes) at room temperature for 1 hour or 4°C overnight, followed by incubation at room temperature in the presence of HRP-linked secondary antibody. Protein was visualized using the ECL system (SuperSignal West Pico chemiluminescence, Pierce).

### Staining of infected cells with Rhodamine B

For labeling with Rhodamine B, ∼2×10^7^ cells were pelleted and resuspended in PBS containing 11 mM glucose and 1 mM Rhodamine B and 10 µg/ml Hoechst 33342. Subsequently cells were incubated at 37°C. Cells were washed five times with PBS-glucose and viewed.

## Supporting Information

Table S1 List of 82 proteins known to enter the secretory pathway. Proteins were identified from the literature and annotation, MaxS and MeanS values are shown.
(0.04 MB XLS)

Table S2 Secretome proteins. Listed are the gene IDs, function, MaxS and MeanS values, method by which protein was identified, identification by mitochondrion and apicoplast targeting prediction programs, and original description in Hiller et al. [14].
(0.13 MB XLS)

Table S3 Comparative analysis of the Hiller and Sargeant secretomes. Data were collected from Hiller et al. and Sargeant et al. [14],[17], as well as from new analyses.
(0.06 MB XLS)

Table S4 Exon distribution of secretome genes (% of total).
(0.03 MB XLS)

Table S5 Changes in annotation of syntenic genes in *P. berghei, P. yoelii, P. chabaudi*, and *P. vivax.*
(0.04 MB XLS)

Table S6 *piggyBac* insertion sites. Strains synthesizing PF13_0317-GFP and PFA0210c-GFP were cloned by limiting dilution and genomic DNA of cloned parasites was isolated. DNA was digested with Sau3AI and self-ligated. Resulting circular DNA was amplified by PCR, and the resulting DNA fragments were cloned into the vector pGEM-T. Inserts were sequenced and insertion sites determined using PlasmoDB.
(0.06 MB XLS)

Table S7 Primers used for amplification of genes and gene fragments.
(0.04 MB XLS)

Figure S1 Anti-GFP immunoblot analysis of the stable *piggyBac* cell lines. Shown are the uncloned parental cell lines. For each immunoblot the position and size of the markers used are indicated on the left. Underneath are listed the molecular weight of each full-length protein (including the signal sequence). For two proteins, indicated by an asterisk, the majority of the protein was detected as free GFP. Parasites were harvested from erythrocytes and lysed as described in the material and methods sections. After separating the proteins by SDS-PAGE and transfer to nitrocellulose, GFP was detected using mono- or polyclonal antibodies directed against GFP.
(1.73 MB TIF)

Figure S2 Comparison of expression profiles of PFC0435w (left) and PF14_323 (calmodulin; right). Top panels show expression levels in *P. falciparum* strains 3D7, DD2 and HB3 with time points taken at every hour. Middle panels represent expression levels in the same three strains synchronized using either sorbitol or temperature, while bottom panels represent level of expression relative to entire genome. Images were taken from PlasmoDB (www.plasmoDB.org; [45]). Transcription data in top panels is based on experiments described in [46],[47], data in middle and bottom panels are derived from [48].
(3.85 MB PDF)

Figure S3 Protein levels of PFC0435w-GFP under control of the calmodulin promoter during the intraerythrocytic life cycle. Parasites expressing PFC0435w-GFP were synchronized by floatation on Percoll and protein was extracted at the times (in parentheses) indicated on top as described in the material and methods. Proteins were separated by SDS-PAGE and after transfer to nitrocellulose, the fusion protein was visualized with a monoclonal anti-GFP antibody. Indicated on the left are the positions and sizes of the markers. On top right is indicated the position of the full-length PFC0435w-GFP fusion and on the bottom right the position of free GFP. T/ES-Trophozoite/early schizont, S-Schizont.
(0.97 MB TIF)

Video S1 Movement of PFC0435c in erythrocyte. Erythocytes infected with *P. falciparum* expressing TVN-JP1-GFP were imaged on a spinning disc confocal microscope. Images were collected every second with an exposure time of 180 ms for thirty seconds. Movie displays five frames per second, and thus is sped up five-fold from real time.
(1.06 MB AVI)
