# Correction: The Malaria Secretome: From Algorithms to Essential Function in Blood Stage Infection

**DOI:** 10.1371/annotation/2b000375-e083-46ed-a44a-ff297e6c37d0

**Published:** 2008-06-27

**Authors:** Christiaan van Ooij, Pamela Tamez, Souvik Bhattacharjee, N. Luisa Hiller, Travis Harrison, Konstantinos Liolios, Taco Kooij, Jai Ramesar, Bharath Balu, John Adams, Andrew P. Waters, Chris J. Janse, Kasturi Haldar

The 11th and 12th authors' names were misspelled. The correct names read: Andrew P. Waters and Chris J. Janse

