# Supplementary figures and images for: The Malaria Secretome: From Algorithms to Essential Function in Blood Stage Infection
Source: PLoS Pathog. 2008 Jun 13;4(6):e1000084. doi: 10.1371/journal.ppat.1000084 (PMC2408878; doi:10.1371/journal.ppat.1000084)

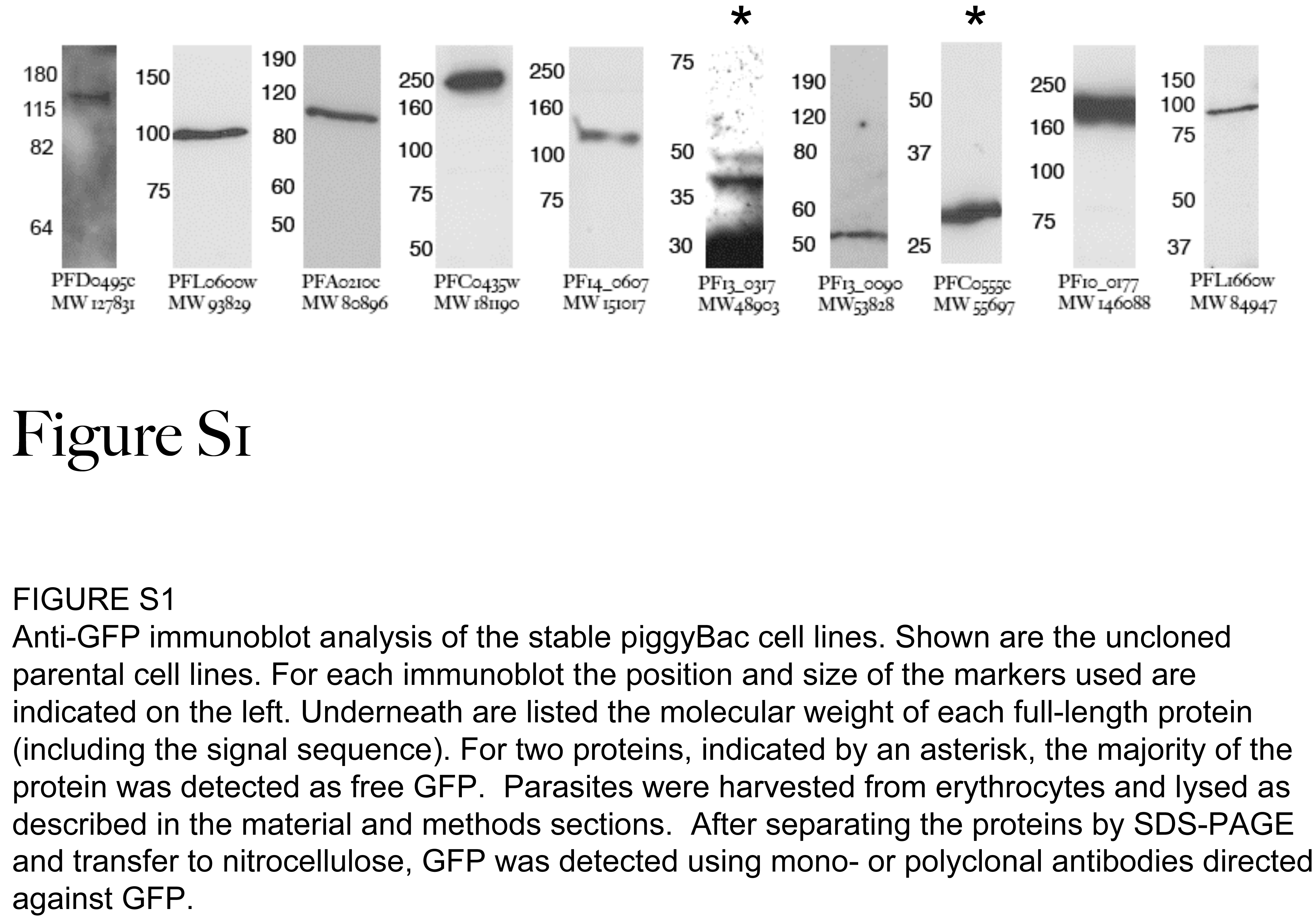

Supplement: Figure S1 — Anti-GFP immunoblot analysis of the stable piggyBac cell lines. Shown are the uncloned parental cell lines. For each immunoblot the position and size of the markers used are indicated on the left. Underneath are listed the molecular weight of each full-length protein (including the signal sequence). For two proteins, indicated by an asterisk, the majority of the protein was detected as free GFP. Parasites were harvested from erythrocytes and lysed as described in the material and methods sections. After separating the proteins by SDS-PAGE and transfer to nitrocellulose, GFP was detected using mono- or polyclonal antibodies directed against GFP. (1.73 MB TIF) [file ppat.1000084.s008.tif]

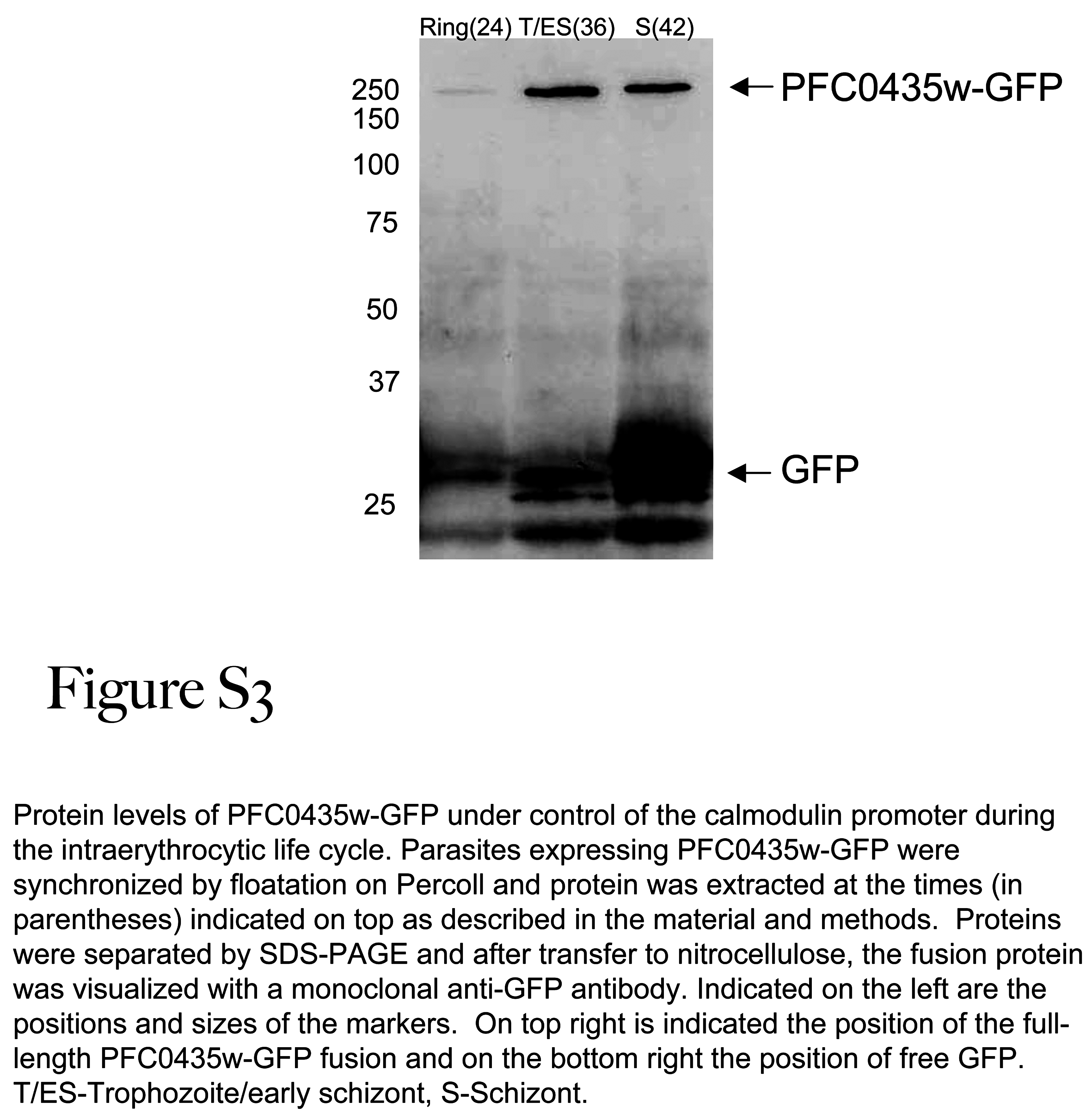

Supplement: Figure S3 — Protein levels of PFC0435w-GFP under control of the calmodulin promoter during the intraerythrocytic life cycle. Parasites expressing PFC0435w-GFP were synchronized by floatation on Percoll and protein was extracted at the times (in parentheses) indicated on top as described in the material and methods. Proteins were separated by SDS-PAGE and after transfer to nitrocellulose, the fusion protein was visualized with a monoclonal anti-GFP antibody. Indicated on the left are the positions and sizes of the markers. On top right is indicated the position of the full-length PFC0435w-GFP fusion and on the bottom right the position of free GFP. T/ES-Trophozoite/early schizont, S-Schizont. (0.97 MB TIF) [file ppat.1000084.s010.tif]
