# Supplementary material for: The Malaria Secretome: From Algorithms to Essential Function in Blood Stage Infection
Source: PLoS Pathog. 2008 Jun 13;4(6):e1000084. doi: 10.1371/journal.ppat.1000084 (PMC2408878; doi:10.1371/journal.ppat.1000084)
Supplement: Figure S2 — Comparison of expression profiles of PFC0435w (left) and PF14_323 (calmodulin; right). Top panels show expression levels in P. falciparum strains 3D7, DD2 and HB3 with time points taken at every hour. Middle panels represent expression levels in the same three strains synchronized using either sorbitol or temperature, while bottom panels represent level of expression relative to entire genome. Images were taken from PlasmoDB (www.plasmoDB.org; [45]). Transcription data in top panels is based on experiments described in [46],[47], data in middle and bottom panels are derived from [48]. (3.85 MB PDF) [file ppat.1000084.s009.pdf]

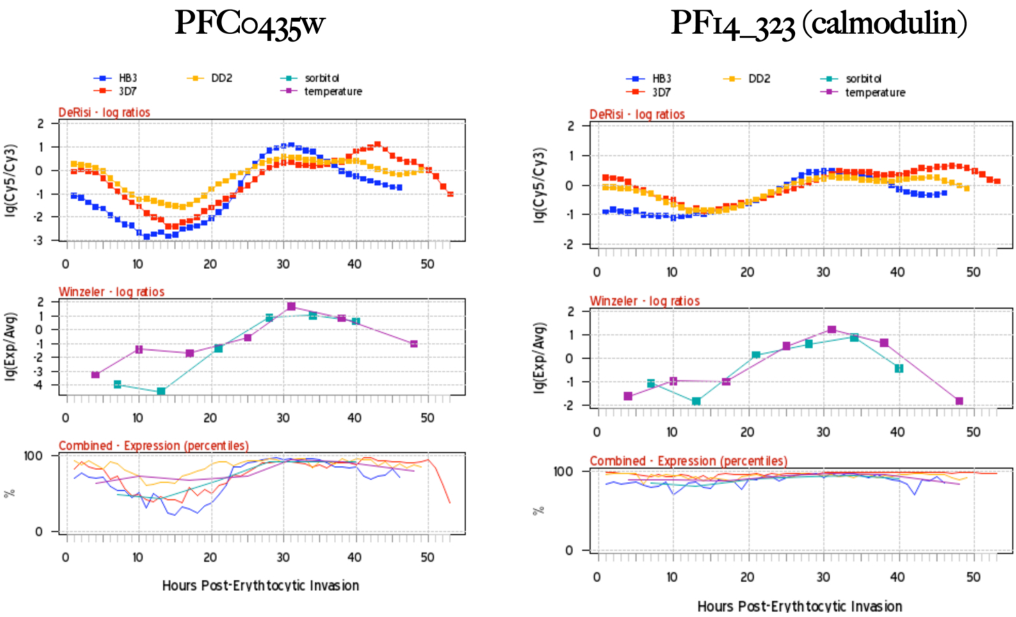

Figure S2
Comparison of expression profiles of PFC0435w (left) and PF14_323 (calmodulin; right). Top panels show expression levels in *P. falciparum* strains 3D7, DD2 and HB3 with time points taken at every hour. Middle panels represent expression levels in the same three strains synchronized using either sorbitol or temperature, while bottom panels represent level of expression relative to entire genome. Images were taken from PlasmoDB (www.plasmoDB.org; [45]). Transcription data in top panels is based on experiments described in [46,47], data in middle and bottom panels are derived from [48].

45. Bahl A, Brunk B, Crabtree J, Fraunholz MJ, Gajria B, et al. (2003) PlasmoDB: the Plasmodium genome resource. A database integrating experimental and computational data. Nucleic Acids Research 31: 212-215.
46. Bozdech Z, Llinas M, Pulliam BL, Wong ED, Zhu J, et al. (2003) The transcriptome of the intraerythrocytic developmental cycle of Plasmodium falciparum. Plos Biology 1: E5.
47. Llinas M, Bozdech Z, Wong ED, Adai AT, DeRisi JL (2006) Comparative whole genome transcriptome analysis of three Plasmodium falciparum strains. Nucleic Acids Research 34: 1166-1173.
48. Le Roch KG, Zhou Y, Blair PL, Grainger M, Moch JK, et al. (2003) Discovery of gene function by expression profiling of the malaria parasite life cycle. Science 301: 1503-1508.
